# Dr. Walker, on the Bony Covering of the Brain

**Published:** 1805-10-01

**Authors:** John Walker

**Affiliations:** Salisbury Square


### *To the Editors of the Medical and Physical Journal.*

Gentlemen,

IN the paper which I sent you a few days since, on the Case of Malformation of the Heart, by John Ring, I offered you some ideas on that important organ. Certain purposes of it are obvious, and beautifully adapted is its structure for the accomplishing of those purposes; yet, while the tyro of later times at once becomes acquainted with the grand functions of this hydraulic machine, how naturally must he be led to consider the whole medical world, previous to the days of Harvey, as composed only of empirics! Must not their whole art have been founded upon experience alone? Indeed, to us, who have had revealed the discoveries made in the animal economy, of streams sanguiferous, lymphatic, &c. our wonder might be excited at the shrewd conjectures of the ancients, totally unacquainted even with the use of the roots (lacteals) whence the waste of the system is repaired; with these, conducting chyle towards the heart; with this, propelling it to all parts of the system—our wonder might be much excited, did we not feel that the most happy application of remedies to the disordered frame has been entirely derived from empiricism. On the *modus operandi* of them, the observation may be trite, we can only make conjectures.

It

It has lately occurred to me, that the obvious use of a bony covering to the brain, a guard against external injuries, is not the only one; that it is also a defence against continual attacks from within, on the grand organ of intelligence and of life. A firm case, different from pericardium, dura mater, or any other yielding tunical covering, seems to me essential to its defence against irregularities in the circulation. The pressure on the brain is continually changing; the contents of the cranium swell gently under ordinary expiration, and are diminished under inspiration. In this there may be a convenient keeping up of the intestine motion, necessary to the living parts, which could not so well have been effected here by strong circulation, nor by muscular motion, as in the other parts of the body. The contents of the cranium also swell, and the pulse quickens under watching and exercise, till at length the refection of sleep is solicited. The organs of the senses and muscles of voluntary motion relax; there is no longer that compression on the sanguiferous system of vessels on the trunk and in the limbs; the blood, consequently, retires from the head, and the brain loses the pressure necessary to a state of wakefulness. Venous sinusses are formed in the head to such an extent as to contain, it is supposed, one-tenth of the whole mass of blood in the body. Into these the veins, arising from the brain, pour their contents, in rather a retrograde direction, which may prove a guard against the effects of reaction, from any inordinate interruption of the venous current at the right side of the heart; any interruption beyond the ordinary slight one at the time of the systole of the auricles. But trifling must be the effect on the brain of such reaction or interruption of the venous stream, in comparison of what must arise from increased action of the heart. When this is produced by any morbid cause, by excess of any kind, by sudden passion, how direct must be the attack upon the brain! Were there not a bony case at such a time, to keep every part of the medullary mass firm in its place, would there not be rupture of delicate parts, interruption of their ineffably and incomprehensibly curious functions or intestine motions? Would not intellect become immediately deranged? Would not death soon close the scene? Other parts of the body may often vary in their volume, may occasionally become plump or tabid, must diminish in declining life; but under all these varieties, will not the contents of the whole cranium continue much much the same in quantity by the pressure of the circumambient air, through their being contained in a cavity of bone?

On this subject I offer you my first ideas, well aware that they cannot be considered correct *a la lettre*, or literally exact, seeing that many enjoy good health after the application of the trephine; which does away the supposed necessity of pressure of the atmosphere keeping the cranium filled: yet from the circumstance of its being requisite for these persons to lead a more guarded life than before, I cannot abandon the idea that a bony cavern for the brain is necessary, to keep it guarded against such irregularities of the circulation, more particularly as other parts bear it with impunity.

By giving this paper a place in the Journal, it may perhaps induce some of your Correspondents to transmit you several curious and valuable observations on the head and heart.

Yours, respectfully,

JOHN WALKER.

*Salisbury Square*, 21, *iv*, 1805.

